# Bribery games on inter-dependent regular networks

**DOI:** 10.1038/srep42735

**Published:** 2017-02-16

**Authors:** Prateek Verma, Anjan K. Nandi, Supratim Sengupta

**Affiliations:** 1 Department of Physical Sciences, Indian Institute of Science Education and Research Kolkata, Mohanpur, 741246, India

## Abstract

We examine a scenario of social conflict that is manifest during an interaction between government servants providing a service and citizens who are legally entitled to the service, using evolutionary game-theory in structured populations characterized by an inter-dependent network. Bribe-demands by government servants during such transactions, called harassment bribes, constitute a widespread form of corruption in many countries. We investigate the effect of varying bribe demand made by corrupt officials and the cost of complaining incurred by harassed citizens, on the proliferation of corrupt strategies in the population. We also examine how the connectivity of the various constituent networks affects the spread of corrupt officials in the population. We find that incidents of bribery can be considerably reduced in a network-structured populations compared to mixed populations. Interestingly, we also find that an optimal range for the connectivity of nodes in the citizen’s network (signifying the degree of influence a citizen has in affecting the strategy of other citizens in the network) as well as the interaction network aids in the fixation of honest officers. Our results reveal the important role of network structure and connectivity in asymmetric games.

An interaction between a corrupt officer demanding a bribe and a citizen seeking a service provide an ideal example of a social conflict scenario. Bribes of this nature, where the citizen is legally entitled to the service sought, are called harassment bribes^1^. This is in contrast to collusive bribes that are paid by an individual or group to illegally get preferential access to a service. Harassment bribes constitute a pervasive form of corruption in many developing countries and while they affect people across all economic strata, low-income individuals are hit the hardest. Hence, proposals to reduce harassment bribery are a matter of great public interest. A few years ago, Basu suggested^1^ that significant reduction in incidents of harassment bribery could be achieved if only the corrupt officers (bribe-takers), but not the bribe-paying citizens, were penalized for taking bribes. Basu argued that such an asymmetry in penalty would induce the bribe-giving citizens to become whistle-blowers against the corrupt officials thereby increasing the likelihood of prosecution of such officials, leading to an overall reduction in cases of harassment bribery. Since Basu’s controversial proposal, several experimental^2^,^3^ and theoretical papers^4^,^5^,^6^,^7^ using conventional game-theory have attempted to elucidate the consequences of asymmetric penalty on harassment bribery. Evolutionary game theory^8^,^9^,^10^ has been profitably used to study a variety of social conflict scenarios^9^,^11^,^12^,^13^,^14^,^15^,^16^,^17^,^18^.

Recently, a lot of attention^19^,^20^ has been focused on understanding the effect of punishment on deterring crime and enabling the spread of cooperation. Helbing *et al*.^21^ explored the circumstances in which cooperators who punish defectors (“moralists”) can spread in a spatially structured population that allows for segregation of moralists from other more exploitative strategies. Short *et al*.^22^,^23^ found that the presence of informants (who report a crime) among cooperators as well as defectors in an evolutionary public goods game can lead to substantial reduction in criminal behaviour. Szolniki and Perc^24^ analyzed the dynamical evolution of different strategies in a spatial public goods game that includes a strategy that combines rewarding cooperators with punishing defectors in addition to simpler strategies that include rewarders, punishers and defectors. Surprisingly, they found that the combined strategy is less likely to prevail. The role of anti-social punishment in thwarting the spread of cooperation has also been explored^25^ though it has been subsequently shown that rewarding of anti-social behaviour by defectors cannot eliminate corruption in spatially structured populations that allow for aggregation of similar strategies provided cooperative behaviour is also rewarded^26^.

It is in the spirit of this rich and diverse literature on crime and punishment that we address the issue of bribery in structured populations. The problem of harassment bribery is ideally suited to be analyzed using evolutionary game theory since it presents a well-defined scenario of social conflict where the interests of the principal players are at cross-purposes. Unlike conventional game theory that seeks to find only equilibrium solutions, evolutionary game theory allows us to better understand the dynamical progression of a population towards equilibrium that takes into account stochastic fluctuations arising from finite population size. It also provides a framework to systematically analyze how the equilibrium state is affected by changes in the initial fraction of different strategies (adopted by both citizens and officers) in the population and other parameters of the model thereby yielding so-called phase diagrams. Such phase diagrams highlight the conditions necessary for honest strategies to prevail. Recently, we carried out a comprehensive study^7^ of the effects of symmetric and asymmetric penalty in *mixed* populations using both deterministic and stochastic evolutionary game theoretic models. Our results revealed the conditions under which the asymmetric penalty scenario was effective in achieving significant reductions in harassment bribery.

A key characteristic of the bribery game in mixed populations was reflected in the ability of any individual (citizen or officer) to interact with and be influenced by any other individual (officer or citizen) in the population. In reality, both citizens and officers are embedded in social networks and form connections within their own groups. Only connected neighbours can influence each other to change their strategies over time. In this paper, we seek to understand the extent to which the structure of the underlying population of citizens and officers affect the outcome of the bribery game.

The nature of the interaction between officers and citizens automatically suggests an inter-dependent network structure with members of each category forming their own networks and interacting with each other via a third network called the interaction network. Unlike in other studies of evolutionary games on inter-dependent networks^27^,^28^,^29^,^30^ that explore the effect of payoff inter-dependency and coupling between two inter-dependent networks, citizens (and officers) do not interact among themselves through their respective networks. The citizen and officer networks are to be considered as replacement networks^31^ used by members to update their strategies by imitating connected neighbours. Hence, there is no overlap between the interaction and replacement graphs. We consider the asymmetric penalty scenario^1^,^6^ where only officers are penalized for taking bribes but citizens do not pay any penalty for giving bribes. We first develop a theoretical model by obtaining time evolution equations for the frequency of each strategy. These equations can be cast in the form of replicator equations with rescaled time. We then carry out stochastic agent-based simulations of the evolution of different strategies on inter-dependent regular networks. We focus on analyzing phase diagrams obtained by varying the bribe amount demanded (*b*) by corrupt officers and the cost of complaining (*t*) incurred by complaining citizens. Our choice is informed by the fact that these two parameters are more easily varied than penalty and prosecution rate, which depend on the efficiency of the criminal justice system.

Another aim is to understand how the connectivity of the underlying networks and asymmetries in the size of the citizen and officer networks affects the spread of honest officers in the population. In general, we find that a structured population helps in the fixation of honest offices over a wider range of parameter space, compared to the mixed population scenario^7^. Intriguingly, we also find that an unconstrained increase in a citizen’s ability to influence the strategies of other citizens in the population (manifest through an increase in the degree of the citizens’ network) does not necessarily aid the proliferation of honest strategies. There exists an optimal range of values of the degree beyond which fixation of honest strategies is no longer favourable. Even though our work focuses on a specific type of social conflict, it nevertheless provides a general framework for understanding asymmetric games on inter-dependent networks.

## Results

### Replicator equations for the bribery game on inter-dependent networks

Evolutionary graph theory provides a useful framework to study the effect of structured population on the dynamics of competing strategies in an evolutionary game. Assuming fitness based selection^32^ Lieberman *et al*.^32^ calculated the fixation probabilities of a single mutant for different types of graphs. Ohtsuki *et al*. (2006)^33^ come up with a simple rule for the evolution of cooperation for a wide variety of social networks. Ohtsuki and Nowak^34^ derived evolution equations for the frequencies of different strategies in a structured population. These equations took the form of the well-studied replicator equation with a transformed payoff matrix and were derived using the pair approximation technique^35^,^36^,^37^,^38^,^39^ under weak selection.

The bribery game^7^ belongs to the category of asymmetric games^40^,^41^ where the competing players have distinct roles. Asymmetry may arise due to environmental factors, genetic variation or simply because players have different social roles. A description of the population structure through separate interaction and replacement graph^31^,^42^ becomes more relevant for asymmetric games like the bribery game where interaction occurs between the players with different social roles but replacement of a strategy is carried out from within players having the same social role. In this section we obtain the replicator equations for the asymmetric bribery game on a graph. Players are assigned either one of the two social roles corresponding to citizens or officers. Following the work of Ohtsuki *et al*.^34^, we employed the pair-approximation under weak selection limit to derive a set of equations describing the time evolution of frequencies of different strategies for a generic asymmetric 2 × 2 game in a structured population for three different update rules (see S8 of “Supplementary Information” for derivations).


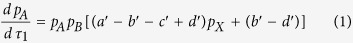



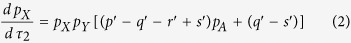


here *p*_*A*_, *p*_*B*_, *p*_*X*_ and *p*_*Y*_ are the frequencies of players with strategies *A, B, X* and *Y* respectively. The value of *τ*_1_ for death-birth, imitation and birth-death update process is 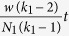, 
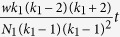
 and 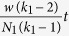 respectively. Similarly the value of *τ*_2_ for death-birth, imitation and birth-death update process is 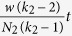, 
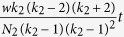
 and 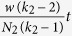 respectively. *N*_1_ and *N*_2_ are the number of players belonging to each social category in the population and *p*_*A*_ + *p*_*B*_ = 1, *p*_*X*_ + *p*_*Y*_ = 1. Population of each category of players is connected through a network of degree *k*_1_ and *k*_2_. Each citizen interacts with *λ*_1_ officers and each officer interacts with *λ*_2_ citizens such that *λ*_1_*N*_1_ = *λ*_2_*N*_2_.

The above eqs (1 and 2) resembles well-studied replicator equation with rescaled time for three different update rules. The dependence on the degree of the two networks is absorbed in the rescaled time and hence they affect the replicator dynamics only indirectly through a change in the equilibration time-scale of the citizen and officer populations. This is distinct from the result obtained for the symmetric game with separate interaction, replacement and common graphs, where update rule affect both time scale and the effective payoff matrix^31^. Deviation from those results can be attributed to the non-existence of a common graph and presence of two replacement graphs in our system. For the 4-strategy bribery game, these equations reduce to (See “Methods” section)


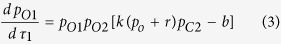



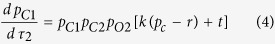


here *p*_*O*1_, *p*_*O*2_, *p*_*C*1_ and *p*_*C*2_ are the frequencies of strategies *O*_1_, *O*_2_, *C*_1_ and *C*_2_ respectively.

### Stochastic Simulations for the five-strategy model on regular networks

**Figure 1:**
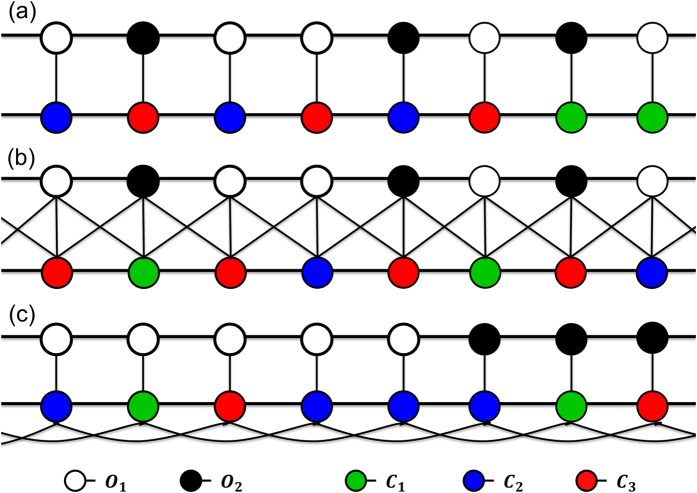
Different types of network construction: (**a**) simplest: *ON* = 2, *CN* = 2, *IN* = 1, (**b**) increased link in interaction network: *ON* = 2, *CN* = 2, *IN* = 3, (**c**) increased link in citizens network: *ON* = 2, *CN* = 4, *IN* = 1. Officer strategies are: *O*_1_ (honest: does not demand bribe) and *O*_2_ (corrupt: demands bribe) and citizen strategies are: *C*_1_ (apathetic: pay silently), *C*_2_ (conscientious: pays but complains) and *C*_3_ (honest: refuse to pay and complains).

The standard replicator dynamics assumes an infinite, homogeneous population with unbiased random pair-wise interactions. The spread of honest officers and conscientious citizens over time is likely to change in structured populations, when the interactions among the players are governed by a nontrivial network topology. Figure 1 shows the topology of the inter-dependent networks for various *IN* and *CN*.

**Figure 2:**
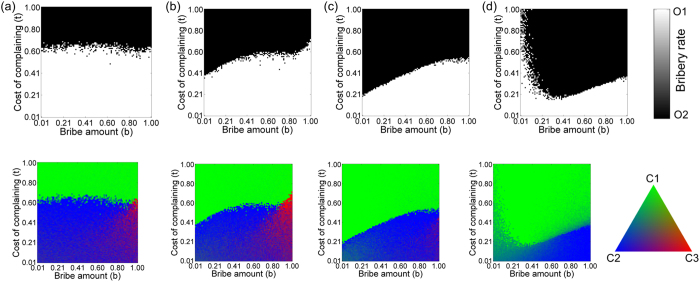
Phase diagrams for officers (upper panel) and citizens (lower panel) for increasing *IN* with parameters *N*_*O*_ = 100, *N*_*C*_ = 100, *ON* = 2, *CN* = 2, *r* = *b* with (**a**) *IN* = 1, (**b**) *IN* = 2, (**c**) *IN* = 4 and (**d**) *IN* = 100. Other fixed parameters are *v* = 1, *c* = 1, *p*_*o*_ = 2, *p*_*c*_ = 0. The region of phase space where honest officers prevail (white) initially increases on increasing the connectivity of the interaction network but subsequently decreases. Honest citizens (C_3_: red) facilitate honest officers to prevail only for high bribe amounts.

Figure 2a shows a phase diagram giving the equilibrium concentrations of different strategies for the simplest inter-dependent network with *CN* = *ON* = 2, *IN* = 1. For a high cost of complaining (*t* ~ 2/3 or more), honest officers failed to survive in the population, and all citizens become *C*_1_ type (pay silently). On the other hand, for low values of *t*, the spread of honest officers in the population is correlated with domination of citizens of type *C*_2_ (pay but complain) in the citizen population. When the bribe amount is large, citizens who pay bribes (*C*_*1*_ and *C*_*2*_) are at a significant disadvantage and hence the presence of honest citizens (*C*_*3*_) aid in the fixation of honest officers in that regime.

We also envisage a scenario in which each citizen can interact with more than one officer. Such scenarios are relevant when each officer provides a distinct service and the citizen needs to access all the services. Since we are considering the nodes to be embedded in space, the connections are restricted to the immediate neighbourhood, and the citizens are connected to the adjacent officers in a regular manner; an example of such a configuration with *IN* = 3 can be seen in Fig. 1b. Each citizen interacts with all the connected officers and accumulates a payoff. The effect of increasing *IN* on the phase diagram can be seen in Fig. 2. As *IN* increases, the phase boundary shifts and the region of phase space where honest officers prevail gradually decreases. For low *IN* (=2), honest officers get eradicated from the population even for relatively lower cost of complaining *t* when the bribe amount *b* is low, but when *b* is high, honest officers can survive at a comparatively higher *t* value due to the presence of honest (*C*_*3*_) citizens (Fig. 2b). As we increase *IN* to 4, we found that the phase boundary is shifted to a lower *t* compared to *IN* = 2 for both low and high value of *b* (Fig. 2c). An increase in *IN* makes an honest (*C*_*3*_) or even a *C*_2_ type citizen more susceptible to be exploited by corrupt officials thereby reducing their payoff and making it easier for such citizens to be eliminated from the population. This tendency persists as we further increase *IN* but for large *IN*, honest officers get fixed in the population for very low *b* and relatively high *t* (Fig. 2d). In case of both low *b* and low *t*, citizens of types *C*_1_ and *C*_2_ do not have much difference in their payoffs; but with an increasing *t, C*_2_ strategy starts becoming comparatively more disadvantageous. As a result, the survival of the honest officers at very low *b* and high *t* becomes correlated with the presence of citizens of type *C*_2_. For *IN* = 100, the maximum possible links a citizen could have in the interaction network, the phase diagram (Fig. 2d) approaches the standard result^7^ (Verma and Sengupta, 2015) obtained by using the replicator dynamics.

**Figure 3:**
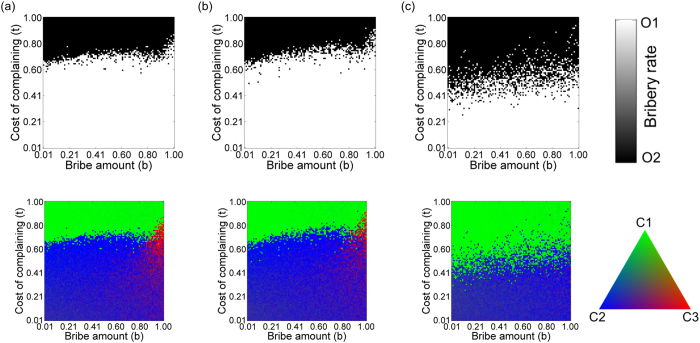
Phase diagrams for officers (upper panel) and citizens (lower panel) for increasing *CN* with parameters *N*_*O*_ = 100, *N*_*C*_ = 100, *ON* = 2, *IN* = 1, *r* = *b* with (**a**) *CN* = 4, (**b**) *CN* = 6 and (**c**) *CN* = 50. Other fixed parameters are *v* = 1, *c* = 1, *p*_*o*_ = 2, *p*_*c*_ = 0. Increasing the degree of the citizen network initially favours fixation of honest officers over a larger area of phase space but subsequent increase is detrimental to their dominance in the population.

Individual citizens while updating their strategy in response to a bribery incident can increase their choices by consulting more than their two nearest neighbours. This can be implemented by increasing the degree *CN* of the nodes in the citizen network. This might increase the chances of apathetic citizens who pay silently (*C*_1_) imitating more honest alternative strategies thereby leading to the spread of such strategies in the population. However, increasing *CN* could also increase the likelihood of apathetic citizens (*C*_1_) influencing their more honest connected but spatially distant counterparts thereby impeding the spread of honest individuals in both citizen and officer populations. To investigate this effect, we varied *CN* to understand how it affects the phase diagram and the fixation probability of honest individuals in the population while keeping the degree of the nodes in the interaction network fixed (*IN* = 1). We find that an initial increase in *CN* allows the fixation of honest individuals over a larger region of parameter space (compare Fig. 2a,b with Fig. 3a,b). The chances of survival of honest officers increases for low as well as high *b* values but the increase is more pronounced for higher values of *b*. Subsequently, the effect of increasing choice (Fig. 3c) is found to be detrimental to the emergence of a bribe-free world.

**Figure 4:**
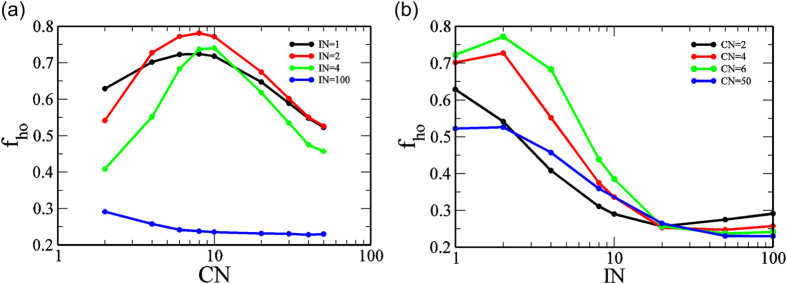
Fraction *f*_*ho*_ of the entire phase space where an honest officer gets fixed plotted (**a**) against *CN* for fixed *IN*. Optimality of *f*_*ho*_ on varying the degree (CN) of the citizen network is observed for low to moderate values of IN. (**b**) *f*_*ho*_ vs *IN* with fixed *CN*. Optimality of *f*_*ho*_ on varying the degree (IN) of the interaction network is observed for moderate values of IN. Other fixed parameters are *N*_*O*_ = 100, *N*_*C*_ = 100, *ON* = 2, *r* = *b, v* = 1, *c* = 1, *p*_*o*_ = 2, *p*_*c*_ = 0.

This intriguing result suggests that there is an optimum value of *CN* for which honest officers can prevail over the largest region of phase space. To quantify this effect we compute *f*_*ho*_, the fractional area of phase space where the honest officers get fixed in the population. Since we varied both *b* and *t* between 0 and 1 with steps of 0.01, we have 10^4^ points in the phase space. To compute *f*_*ho*_, we counted the number of cases where the honest officers get fixed in the population and divided the number by 10^4^. We plotted *f*_*ho*_ against *CN* for different *IN* (Fig. 4a). We found that there is indeed a maximum for *f*_*ho*_ at a moderate value of *CN*, though the maximum is different for different *IN*. Highest *f*_*ho*_ was achieved for *IN* = 2. We also plot *f*_*ho*_ against *IN* while varying *CN* as parameter (Fig. 4b) and found a peak at *IN* = 2 for moderate values of *CN*. The peak disappears at very low or high values of *CN*.

Therefore, we can conclude that a moderate number of connections among the citizens are necessary to promote a bribe-free world. This advantage also reduces significantly when the degree of the officers’ network (*ON*) increases. As we increased *ON*, information about the success of corrupt officers is more widely dispersed resulting in the fixation of corrupt officers in the sub-population (results not shown).

This effect can also be captured by plotting the fixation probability for varying *CN* and *IN* but for a specific value of *b* and *t* that lies near the phase boundary. Since fluctuations are more prominent near the phase boundary compared to a point in the interior of either region of the phase diagram, fixation of honest officers is not always guaranteed for such a point. We choose to show a particular combination of *b*and *t* for which this effect is prominent and find the fixation probability of honest officers for a range of *CN* values after averaging over 100 realizations. We found that at a moderate number of connections among the citizens, the probability of fixation reaches its maxima. The results are depicted in more detail in Figure S1 of Supplementary Information.

**Figure 5:**
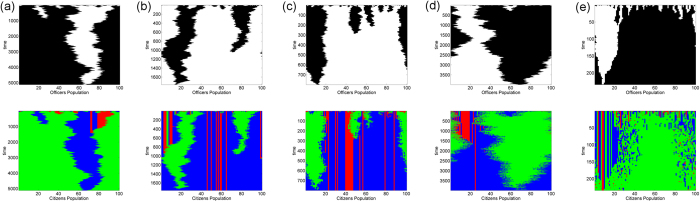
Distribution of strategies in officer’s (top panels) and citizen’s (bottom panels) population over time as the degree of the citizen’s network is increased from (**a**) CN = 2 to (**b**) CN = 4, (**c**) CN = 6, (**d**) CN = 20 (**e**) CN = 50. Population is embedded on a regular linear chain network topology. Each figure in the panel is a result of a single simulation for a fixed parameter set initialized with equal number of different strategy players distributed randomly over their respective networks. X-axis represents the distribution of strategies of players at a particular time. Y-axis (downward direction) is the time axis. Black and white colors represent corrupt and honest officers respectively. Green, blue and red colors are for citizens who pay silently (*C*_1_), pay & complain (*C*_2_) and refuse to pay (*C*_3_) respectively. The values of the parameters for the simulation is *v* = 1, *c* = 1, *p*_*o*_ = 2, *p*_*c*_ = 0, *b* = 0.70,*t* = 0.70, *r* = *b, N*_*O*_ = 100, *N*_*C*_ = 100, *ON* = 2, *IN* = 1.

Figure 5 shows the effect of increasing the citizen’s connectivity (*CN*) on the population dynamics. It is worth noting that though we start with random distribution of strategies, after successive updates over time, corrupt officers (*O*_*2*_) tend to form interacting clusters with apathetic citizens (*C*_*1*_) and honest officers (*O*_*1*_) form clusters with complaining citizens (*C*_*2*_ and *C*_*3*_). This happens because corrupt officers connected with complaining citizens will have lower payoff. These corrupt officers then have a higher probability of imitating their neighbouring honest officer’s strategy. On the other hand corrupt officers’ payoff gets a boost when apathetic citizens silently pay bribes to such officers. This increases the likelihood that a neighbouring honest officer will imitate a corrupt officer during the update process. Except for very high values of *b*, when payoffs of *C*_2_ and *C*_3_ become comparable, honest (*C*_3_) citizens die out very quickly due to their low payoff, and the competition for survival continues between *C*_1_ and *C*_2_ types only. The eventual fixation of a specific strategy in the population depends upon which cluster expands and which one shrinks over time. Expansion of a cluster in the network depends on the dynamics at the boundary where two large clusters meet. In Fig. 5(a) corrupt officers (black) along with their apathetic citizen (green) counterparts in the citizen network take over the entire population by around 5000 generations. As CN increases from 2 to 4, honest officers along with conscientious citizens take over the entire population by 1800 time steps. This trend continues for a while as CN is further increased but is eventually reversed for large CN (Fig. 5e) indicating an optimal range of connectivity of the citizen network is required for bribery to be eradicated. The population update dynamics and the effect of the degree of the nodes in the citizen network on the survival of honest officers (and conscientious citizens) can be understood by analyzing the interactions between the players with different strategies at the boundary of the clusters.

**Figure 6:**
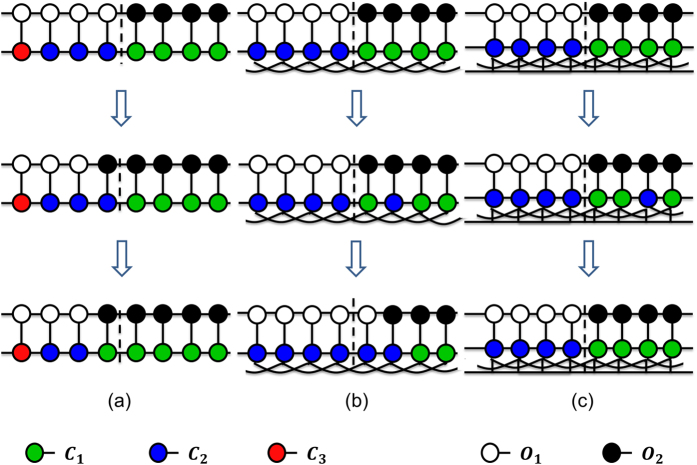
Figures showing how clusters in the officer and citizen networks can grow or shrink over time. (**a**) CN = 2, (**b**) CN = 4, (**c**) CN ≫ 4.

Consider the simplest possible network configuration with *ON* = *CN* = 2, *IN* = 1 (Fig. 6a) and focus on the population structure at a time when clusters have already been formed (*O*_1_ interacting with *C*_2_ or *C*_*3*_ and *O*_2_ interacting with *C*_1_). At the cluster boundary, an honest officer (who acquires a payoff *v* while interacting with *C*_*2*_ or *C*_*3*_) will adopt the strategy of a neighbouring corrupt officer (who acquires a payoff *v + b*) with a probability *b*/[2(*b *+ *p*_*o*_)] (when *r* = *b*). At the same time, *C*_1_ the interacting partner of O_2_ at the boundary will adopt the strategy of a neighbouring *C*_2_ with a probability *b*/[2(*c *+ *t*)] that is greater than the former. Therefore it is more likely that in the next generation, the officers’ strategies do not change, but *C*_1_ changes to *C*_2_. In the next step, this new *C*_2_ when interacting with *O*_2_, pays the bribe and complains. If a successful prosecution happens, *C*_2_ retains her strategy with probability 1 if *b *≥ *t*, also with a high probability of 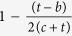 if *b* < *t*. At the same time, *O*_2_adopts the neighbouring *O*_1_ strategy with a probability of 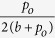. In this process, a cluster of honest officers can increase in size when complaining citizens are also increasing in the corresponding citizen’s network by converting their respective neighbours at the boundary. But if the prosecution does not happen, then the new *C*_2_ could copy the neighbouring *C*_1_ with a probability and the initial situation would be restored.

Alternatively, if at the very first step, *O*_1_ successfully imitates *O*_2_ (middle panel of Fig. 6a), this new *O*_2_ will interact with *C*_2_ in the next generation. If *O*_2_ is not prosecuted, *C*_2_ would end up with a low payoff (*c*−*b*−*t*) and would therefore be more likely to imitate the neighbouring *C*_1_ (bottom panel of Fig. 6a) with a probability 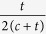. As a result, the clusters for the honest individuals would shrink. These competitions over the growth of the clusters in either network are controlled by the values of *b, t* and *k*.

When the degree of citizen’s network is increased to four (Fig. 6b), the nearest along with next nearest neighbour at the boundary can also imitate complaining citizens with a probability 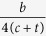 (less by a factor of half compared to the *CN* = 2 case). If this happens, (middle panel of Fig. 6b), then in the next step, it is much more likely that *C*_*1*_ nearest to the cluster boundary also changes to *C*_*2*_ (since 3 out of 4 of her neighbours are *C*_*2*_). That, in turn, would favour the nearest and the next nearest corrupt officers to become honest, one after the other. This shows how the cluster of honest officers and corresponding complaining citizens can increase over time leading to a higher chance (on an average) of honest officers getting fixed in the population.

When the degree of citizen’s network is substantially increased, the likelihood of an apathetic citizen in the interior of a cluster imitating a complaining citizen’s strategy increases but the probability of affecting the corrupt officer at the boundary decreases. Figure 6c depicts one such possible scenario where a citizen in the interior of the cluster of apathetic citizens becomes a complaining one. This leads to reduced payoff for the corresponding corrupt officer. Since that corrupt officer is connected to two other corrupt officers, she cannot change her strategy. Moreover, since the nearest and next-nearest apathetic citizen neighbours at the boundary are not affected, the cluster size does not change. Eventually, the lone complaining citizen in the interior reverts back to an apathetic citizen by imitating one of her connected apathetic neighbours. The change in cluster size can occur when apathetic citizens and corrupt officers proliferate due to imitation of their strategies by their honest counterparts at the boundary of the clusters. This scenario is more likely for large values of CN as manifest in Fig. 5E.

### Scenarios without refund

In most realistic scenarios, it is less likely for the bribe-amount paid by a citizen to be refunded even if the corrupt officer is prosecuted and penalized for the act of taking bribes. We therefore consider a situation when the bribe-giver does not receive any refund (*r* = 0). The phase diagram for various values of *CN* (Figure S2 of Supplementary Information) shows that in such a case, the region of phase space in which honest officers get fixed not only shrinks considerably (compared to Figs 2a and 3a–c), the shape of the phase boundary also changes. A peak in the fraction of phase space containing only honest officers is also observed on varying *CN*. Interestingly, for low *CN* and moderate values of cost of complaining *t*, corrupt officers need to optimize the bribe demand to survive in the population. This is manifest in the form of a V-shaped phase boundary. This can be understood by focusing on the cluster boundary where *O*_2_ is interacting with *C*_1_ and *O*_1_ is interacting with either *C*_2_ or *C*_3_. In either case, *C*_1_ will be replaced by the respective complaining citizen with probability 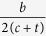. As before, this new complaining citizen (*C*_2_ or *C*_3_) will now interact with the corresponding corrupt officer *O*_2_ and subsequently lodge a complaint. For the expansion of the cluster of honest individuals, this complaint has to be successful implying that the corrupt officer has to be prosecuted. When such a prosecution happens in the presence of a *C*_2_, the corresponding *O*_2_ would copy the *O*_1_ strategy with a probability 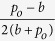. At the same time, *C*_2_ would retain her strategy with a probability 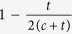. On the other hand, if the complaining citizen is a *C*_3_, these probabilities are 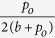 and 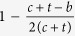 respectively. Comparing the probabilities for *O*_2_ to change, we see that if *b* is high, a *O*_1_−*C*_3_ combination at the cluster boundary grows faster than a *O*_1_−*C*_2_ combination. However, a *O*_1_−*C*_2_ combination performs better for low *b*. These two opposing factors compete in the region of moderate *b*. It is also evident that the probabilities with which *C*_*2*_ and *C*_*3*_ retain their strategies decrease with increasing *t*. The effects of *t* in decreasing these probabilities are less prominent for extreme values of *b* where one of those two opposing factors is dominant. But in the intermediate region, the effect of *t* becomes significant even for comparatively low values of *t*. Thus we get V-shaped phase boundaries.

Figures 2 and 3 suggest that the citizens who refuse to pay (*C*_3_) play an insignificant role, in the equilibrium strategy distribution in most regions of the *b-t* phase space. We, therefore, explored bribery games with (Figure S3) and without (Figure S4) refund by dropping *C*_3_ type citizens from the initial population. The results are similar to the 5-strategy model and here too we observe a peak in the fraction of phase space containing honest officers only for moderate value of *CN*. The honest officers in the equilibrium population occupy a significantly larger region of the parameter space compared to the mixed population case^7^. Since citizens of type *C*_3_ are not present, honest officers do not get any advantage for high *b* and low *t* case, but retain their advantages in the low *b, t* domain for the case without refund.

### Asymmetric populations

**Figure 7:**
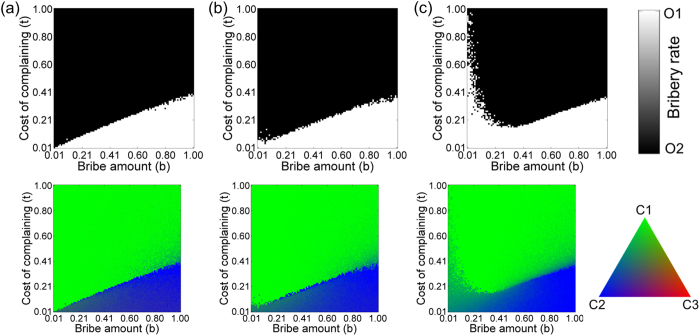
Effect of asymmetry in population of officers and citizens on varying IN. Phase diagrams showing the equilibrium state of officers (upper panel) and citizens (lower panel) when the degree of the interaction network is changed. (**a**) *IN* = 1, (**b**) *IN* = 4 and (**d**) *IN* = 100. Other fixed parameters are: *N*_*O*_ = 100, *N*_*C*_ = 1000, ON = 2, *CN* = 2, *r* = *b, v* = 1, *c* = 1, *p*_*o*_ = 2, *p*_*c*_ = 0.

In the previous model, both the citizens’ and the officers’ sub-populations were of equal sizes. In reality, the citizens’ sub-population is expected to be much larger than the officers’. Keeping this in mind, we investigate the bribery game by considering the citizens’ network to be 10 times larger than that of the officers’ network (*N*_*O*_ = 100, *N*_*C*_ = 1000). Now, even for *IN* = 1, each officer interacts with 10 adjacent citizens, as if the officer has been appointed to serve a locality of 10 people. The result of the game, in the phase space defined by *b* and *t*, is shown in Fig. 7a. In comparison with the previous model (Fig. 2a), the area dominated by honest officers is drastically reduced. Since an officer is playing with multiple citizens and the rate of prosecution is low, if a corrupt officer gets punished while dealing with *C*_2_ or *C*_3_ type citizen, she has ample chance to compensate her loss in payoff by taking bribes from connected *C*_1_ type citizens. On increasing *IN*, honest officers started to get fixed at low *b* (Fig. 7b,c), and as in the case depicted in Fig. 2d, we recover the replicator dynamics result when *IN* = 100 (Fig. 7c).

**Figure 8:**
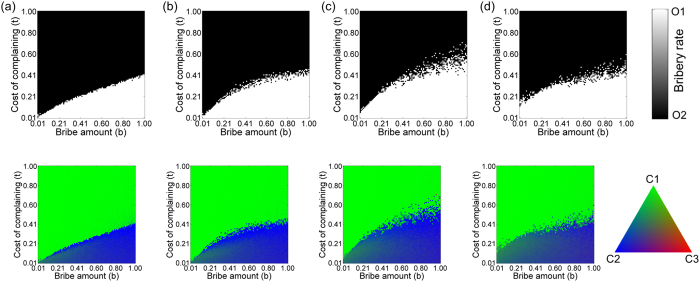
Effect of asymmetry in population of officers and citizens on varying CN. Phase diagrams showing the equilibrium state of officers (upper panel) and citizens (lower panel) when the degree of the citizen network is changed; (**a**) *CN* = 8, (**b**) *CN* = 20, (**c**) *CN* = 50, (**d**) *CN* = 200. Other fixed parameters are: *N*_*O*_ = 100, *N*_*C*_ = 1000, ON = 2, *IN* = 2, *r* = *b, v* = 1, *c* = 1, *p*_*o*_ = 2, *p*_*c*_ = 0.

Increasing *CN* to 4 or 6 would have little effect on the equilibrium concentration of the players. Since a group of 10 citizens interact with a single officer, the payoffs of the citizens are comparatively less variable. To see the effect of increasing *CN*, the degree of the citizens’ network should reach well beyond the group of 10 people. Figure 8 shows the effect of varying *CN* and we can recognize an optimal connectivity of *CN* for which the chances of survival of honest officers reach a maxima, just as in the model discussed earlier. However, the value of *CN* at which the peak occurs is much larger than before for reasons mentioned above. This is also evident by plotting the fraction of *b-t* phase space in which honest officers get fixed in the population vs *CN* and *IN* (see Figure S5 in Supplementary Information). In Figure S6, we show the phase diagram for varying *IN* but fixed *CN* = 50. Comparing Figure S6 with Fig. 8c, it is clear that the *IN* = 2 case is more favourable to honest officers than other IN values.

## Discussion

We explored the effect of network connectivity on the prevalence of bribery in a structured population characterized as an inter-dependent regular network. In general, we find that the spread of corruption is impeded in a structured population in comparison to a mixed population. Honest individuals dominate the population over a larger region of parameter space. However, the equilibrium structure of the citizen and officer populations also depends on the connectivity of the underlying regular networks. Increasing the connectivity of the citizen network beyond a point is counterproductive for the survival of honest officers in certain regions of phase space. The fixation probability of honest officers for points near a phase boundary increases up to a point as a citizen’s ability to influence the behavior of another in the network is increased. Beyond that point, further increase in a citizen’s domain of influence leads to a decrease in the fixation probability of honest officers.

In a networked population, conscientious and apathetic citizens quickly form clusters where they interact with honest and corrupt officers respectively. The payoff structure determines which of these clusters grow and eventually take over the entire population. An asymmetry in the size of the officer and citizen populations ensures that multiple citizens interact with a single officer for a service. An officer therefore accumulates payoffs from multiple interactions and reduction in payoff due to interaction of a corrupt officer with a single or a few honest/conscientious citizens is therefore less pronounced. Under such circumstances, eradicating bribery becomes a lot more difficult. Increase in the degree of the citizen network can have a positive impact in reducing bribery over larger region of parameter space provided the increase enables citizens to influence those that lie beyond their own cluster that’s interacting with one designated officer. In the absence of refunds, payoffs to conscientious citizens decrease resulting in bribery being sustained over a larger region of parameter space. When the amount of bribe demanded is high, honest citizens do better compared to the conscientious citizens implying whistleblowing is not effective while the reverse is true when the bribe demanded is low. However, for intermediate values of b and t, apathetic citizens dominate resulting in a V-shaped phase boundary suggesting that corruption is more likely to be sustained if the bribe-demand is optimized by corrupt officials.

As in previous studies^6^,^7^ we too find that lowering the cost of complaining can have a significant impact on reducing corruption. Governments can facilitate submission of online complaints that can bring down the cost of whistleblowing, thereby increasing accountability and efficiency of grievance redressal in the asymmetric penalty scenario. However, our network-based analysis also indicates that the effectiveness of reducing the cost of complaining depends not just on the bribe amount but also crucially on the connectivity of each of the inter-dependent networks.

Even though, we focus on the bribery game, our analysis provides a general framework for studying *asymmetric* games on inter-dependent networks and reveals the importance of connectivity of each of the three networks on the underlying social conflict dynamics. The asymmetry arises due to distinct roles ascribed to citizens and officers in our model and yield behaviour that provides an interesting contrast to the analysis^30^ of PD games on inter-dependent networks that is most relevant to our work. As in that study, we also find the coupling between the two networks plays an important role in the prevalence of corruption. However, in contrast to that study where the independence of the two networks vanished as the probability of coupling increased to 1 (IN = 1 in our model), the asymmetric nature of the interactions in our model ensures independence of the two networks even when the connectivity of the two networks is increased beyond IN = 1. This allows us to explore the effect of the degree of the interaction network on the persistence of corruption.

Future directions involve studying the consequences of evolutionary dynamics in more complex networks of citizens and officers. We also feel our work can inform the design of more effective sociological experiments to understand the evolution of corrupt strategies and help in formulating policies that reduce harassment bribery. In summary, our work reveals the significant difference in the evolutionary dynamics of honest and corrupt strategies in structured and mixed populations and highlights the importance of optimal network connectivity in reducing incidents of harassment bribery in structured populations.

## Methods

### Theoretical Analysis

Players are assigned either one of the two social roles corresponding to citizens or officers. Each category of players can adopt one of the two distinct strategies available to them. Officers may choose from strategies *A* and *B*. while citizens can choose from strategies *X* and *Y*. The payoff matrix of the game is given by


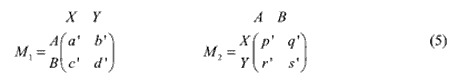


Citizens interact only with officers and vice versa and can be replaced only by other citizens who are connected neighbours of the focal player. We study three different update rules: (a) death-birth (b) imitation and (c) birth-death. In ‘death-birth’ process a random individual is chosen for death, neighbouring individuals then compete to occupy the empty node with probability proportional to the fitness of the competing individuals. According to imitation update rule, a random individual (focal player) imitates one of its neighbours’ (role model) strategies with probability proportional to the fitness of the neighbour (role model). In ‘birth-death’ an individual is selected for reproduction proportional to fitness and replaces one of its randomly chosen neighbours.

For the bribery game, strategies A and B correspond to honest (O_1_) and corrupt (O_2_) officers while X and Y correspond to apathetic (pay silently: C_1_) and conscientious (pay and complain: C_2_) citizens respectively. Elements of payoff matrix for a four strategy bribery game are given by: *a*′ = *v, b*′ = *v, c*′ = *v *+ *b, d*′ = *v *+ *b* − *k*(*p*_*o*_* *+ *r*), *p*′ = *c, q*′ = *c* − *b, r*′ = *c* and *s*' = *c* − *b* − *t *+ *k*(*r* − *p*_*c*_). Where the citizens’ fixed payoff *c* can be thought of as the cost of the service, officers’ fixed payoff *v* as the salary of the officer and *b* (<*c*) as the amount of bribe demanded by the corrupt officers. To lodge a complaint to the respective authority costs the citizen an amount *t*. The probability of prosecution is given by*k* (0 < *k* < 1); *r* is the bribe-amount refunded to the citizen if the guilty officer is prosecuted for taking bribes; while *p*_*o*_ is the amount penalty paid by a corrupt officer when prosecuted and *p*_*c*_ is the amount by which a citizen is penalized for giving bribes.

### Stochastic Simulations

We embed the individual players on regular networks where the players are restricted to interact with their connected neighbours only as depicted in Fig. 1 where the connected neighbours are linked by solid black lines. The specific nature of the bribery game leads to a formulation in terms of an inter-dependent network consisting of three sub-networks. These are citizen and officer networks that specify the links (black lines) between citizens (represented by red, green and blue circles in Fig. 1) and officers (represented by black and white circles in Fig. 1) and an interaction network that specifies how each citizen interacts with officers. The citizen/officer networks determine how a citizen/officer can update her strategy by imitating the strategy of a connected neighbour in her network following a bribery game with a member of the officer/citizen network. In this article, we consider each of the three sub-networks to be regular networks with a fixed degree.

As the simplest form of a model network for both citizens and officers, we consider the 1-d lattice chain with periodic boundary condition, where each site is occupied by a player and each player is connected to a fixed number of adjacent nearest neighbours (*CN* for the citizens’ and *ON* for the officers’ network respectively). For the interaction network, we considered each citizen to be connected with *IN* officer(s) (for example, IN = 1, 3 in Fig. 1a,b respectively). We carried out simulations for different degrees of citizens’, officers’ and interaction sub-networks.

Initially, strategies were randomly distributed among the players. Each officer is assigned any one of the two available strategies *O*_1_ (honest: does not demand bribe) and *O*_2_ (corrupt: demands bribe) with a probability of 1/2. Similarly, each citizen is assigned one of the three available strategies *C*_1_ (apathetic: pay silently), *C*_2_ (conscientious: pays but complains) and *C*_3_ (honest: refuse to pay and complains) with a probability of 1/3. The payoffs to each strategy resulting from interactions between officers and citizens are depicted in Figure S7 of the “Supplementary Information” and the game can be represented by the corresponding payoff matrix


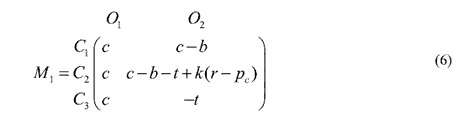



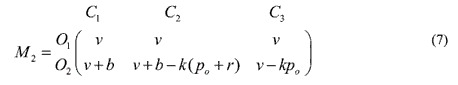


We consider an asymmetric penalty scenario in which an officer is penalized by an amount of *p*_*o*_ when caught taking a bribe, but the citizen paying the bribe receives no penalty, *p*_*c*_ = 0. We study scenarios both with and without refund. In the former case, the citizen gets back her bribe-amount *b* as refund *r* (=*b*) while in the latter case, *r* = 0. To generate a phase diagram, we vary both the bribe amount *b* and the cost of complaining *t* between 0 to 1 in steps of 0.01 and for each combination of *b* and *t* keeping the same initial configuration of strategies in both the networks.

In our model, the citizen and officer sub-populations have sizes *N*_*C*_ = 100, 1000 and *N*_*O*_ = 100 respectively. Strategies are distributed randomly among the citizens and the officers. In a single round, each citizen plays the bribery game with all of her officer neighbours in the interaction network and accumulates the payoffs. Then, following a parallel update rule, the focal citizen randomly selects one of her connected neighbour in the citizens’ network and compares her own payoff with that of the selected individual’s. If the focal individual’s payoff *π*_*C*_ is found to be greater than or equal to the selected individual’s payoff 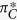, the focal individual’s strategy remains the same. Otherwise she adopts the strategy of the selected individual with a probability 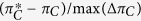, where 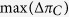 is the maximum possible payoff difference between any two citizens. In a similar manner, officers payoffs also get accumulated and following a similar update rule, the focal officer randomly selects one of her neighbour in the officers’ network and if her payoff 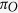 is smaller than that of the selected officer 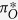, she copies her strategy with a probability 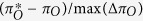, where 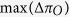 again being the maximum possible payoff difference between any two officers. The steps are repeated for 10^10^ generations or until any one of the officers’ strategies gets fixed, whichever is earlier.

## Additional Information

**How to cite this article:** Verma, P. *et al*. Bribery games on inter-dependent regular networks. *Sci. Rep.* **7**, 42735; doi: 10.1038/srep42735 (2017).

**Publisher's note:** Springer Nature remains neutral with regard to jurisdictional claims in published maps and institutional affiliations.

## Supplementary Material

Supplementary Information
