# Supplementary material for: Bribery games on inter-dependent regular networks
Source: Sci Rep. 2017 Feb 16;7:42735. doi: 10.1038/srep42735 (PMC5311942; doi:10.1038/srep42735)
Supplement: Supplementary Information [file srep42735-s1.pdf]

Supplementary Information

Bribery games on inter-dependent regular networks

Prateek Verma, Anjan K. Nandi and Supratim Sengupta

Department of Physical Sciences, Indian Institute of Science Education and Research Kolkata, Mohanpur-741246, India.

Figure S1: Fixation probabilities for players with different strategies plotted against CN for a particular combination $b=0.70$, $t=0.70$ with a) $IN=1$ and b) $IN=2$. Other fixed parameters are $N_O=100$, $N_C=100$, $ON=2$, $r=b$, $v=1$, $c=1$, $p_o=2$, $p_c=0$.

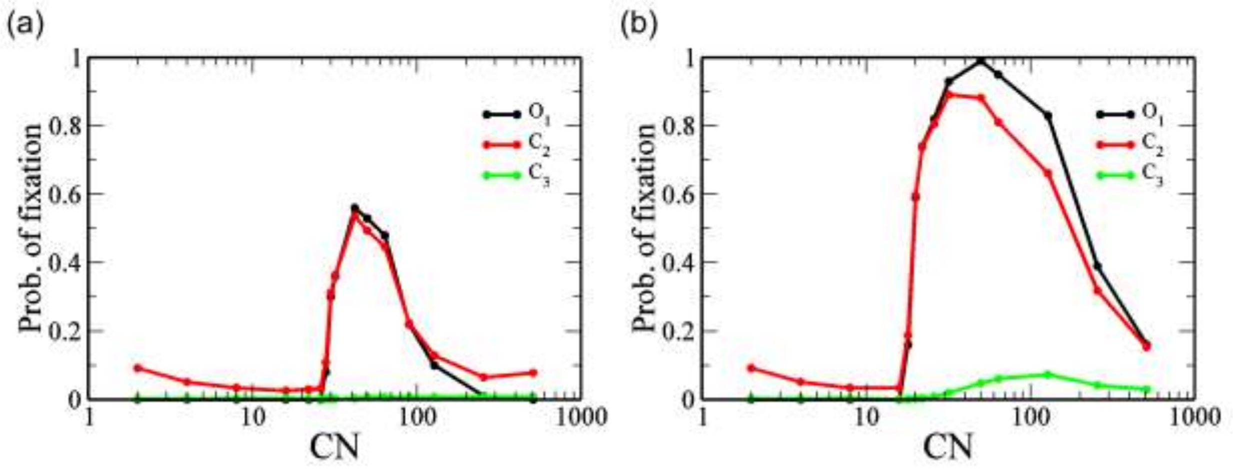

Figure S2: Phase diagrams for officers (upper panel) and citizens (lower panel) for increasing CN without refund $r=0$, for a) $CN=2$, b) $CN=4$ c) $CN=6$ and d) $CN=50$. Other fixed parameters are $N_O=100$, $N_C=100$, $ON=2$, $IN=1$, $v=1$, $c=1$, $p_o=2$, $p_c=0$.

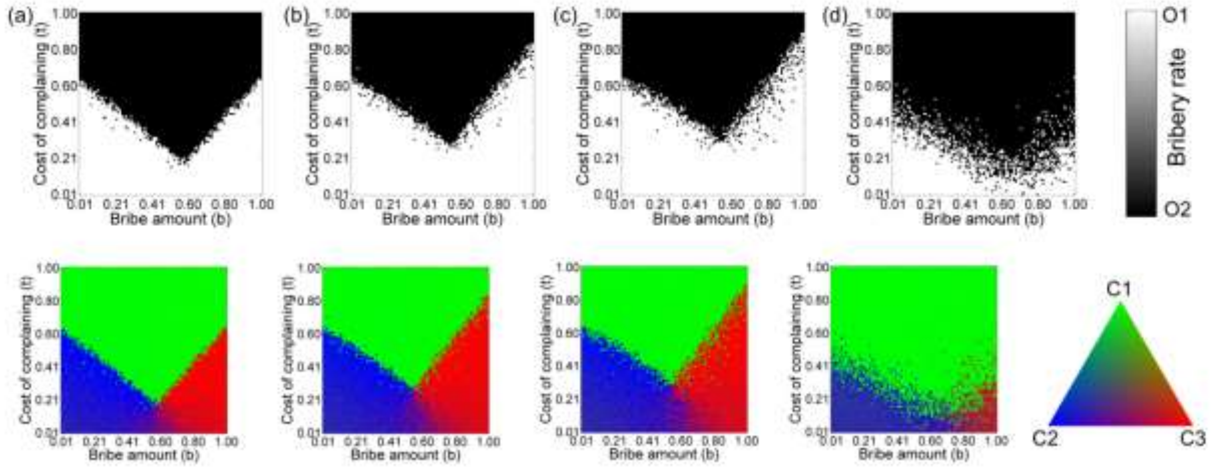

Figure S3: 4 strategy model: Phase diagrams for increasing CN with refund $r = b$, for a) $CN = 2$, b) $CN = 4$ c) $CN = 6$ and d) $CN = 50$. Other fixed parameters are $N_O = 100$, $N_C = 100$, $ON = 2$, $IN = 1$, $v = 1$, $c = 1$, $p_o = 2$, $p_c = 0$.

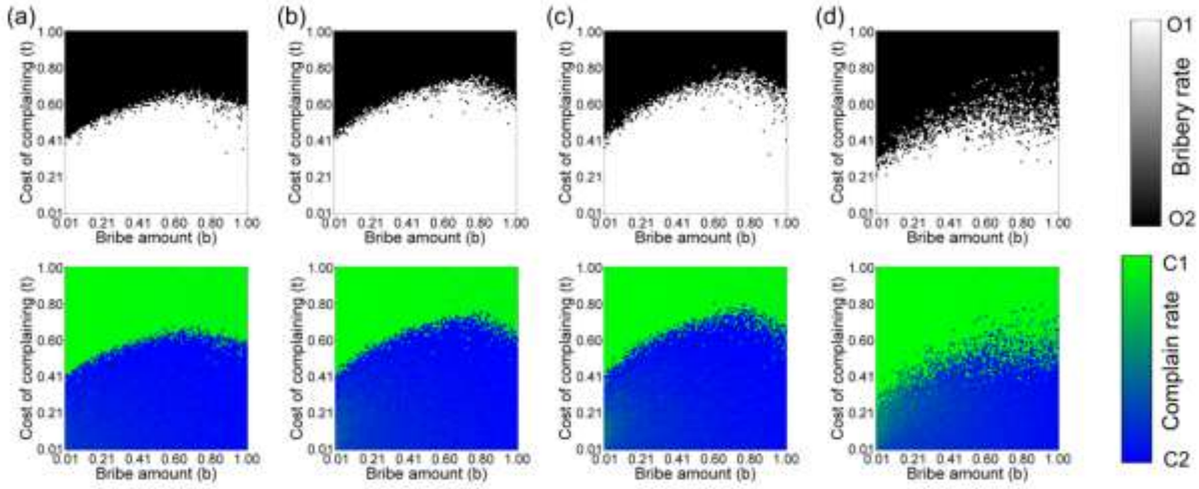

Figure S4: 4 strategy model: Phase diagrams for officers (upper panel) and citizens (lower panel) for increasing CN without refund $r = 0$, for a) $CN = 2$, b) $CN = 4$ c) $CN = 6$ and d) $CN = 50$. Other fixed parameters are $N_O = 100$, $N_C = 100$, $ON = 2$, $IN = 1$, $v = 1$, $c = 1$, $p_o = 2$, $p_c = 0$.

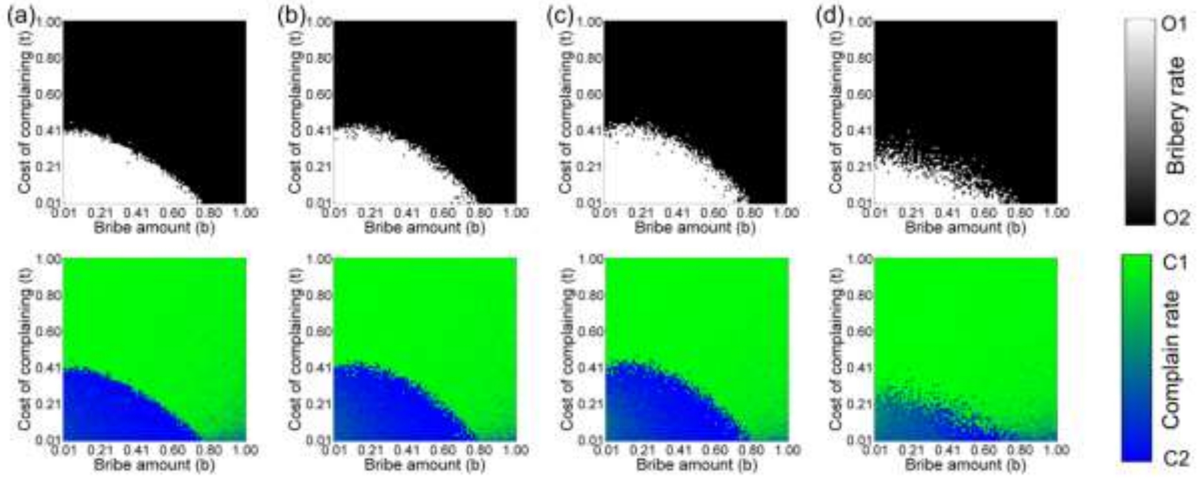

Figure S5: Fraction of points f_{ho} in the entire phase space where an honest officer gets fixed plotted a) against CN for different IN values, b) against IN for different CN values. Other fixed parameters are $N_O = 100$, $N_C = 1000$, $ON = 2$, $r = b$, $v = 1$, $c = 1$, $p_o = 2$, $p_c = 0$.

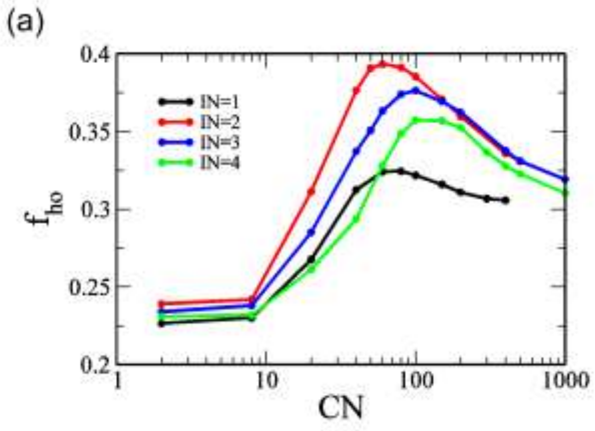

Figure S6: Phase diagrams for officers (upper panel) and citizens (lower panel) for increasing IN with parameters $N_O = 100$, $N_C = 1000$, $ON = 2$, $CN = 50$ $CN = 50$, $r = b$ with a) $IN = 1$, b) $IN = 4$ and c) $IN = 100$. Other fixed parameters are $v = 1$, $c = 1$, $p_o = 2$, $p_c = 0$.

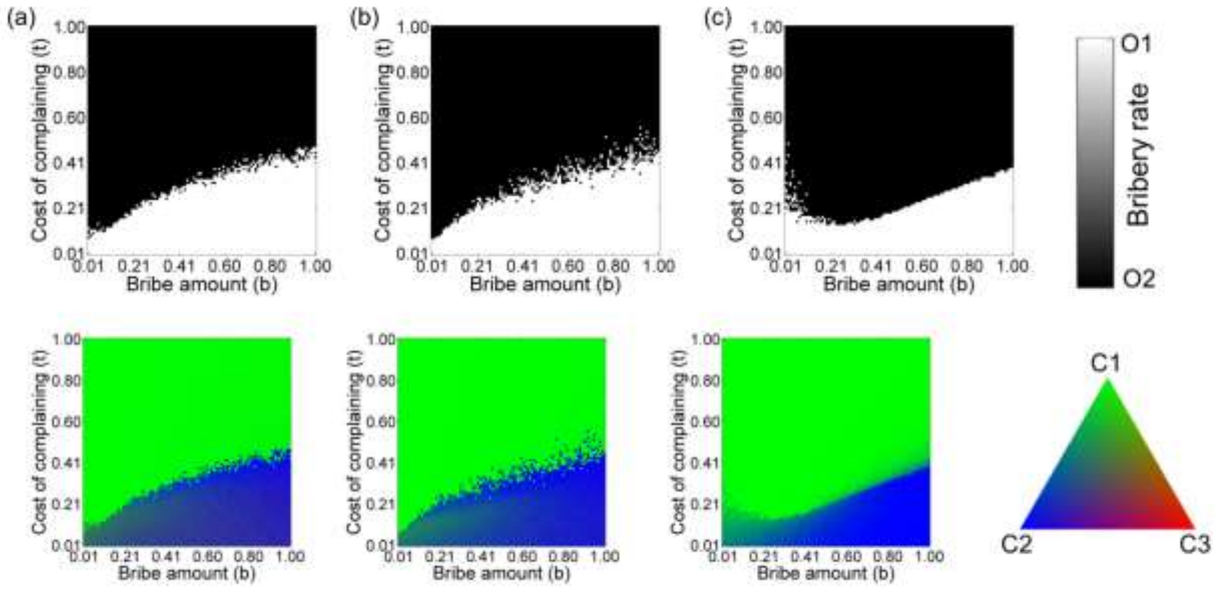

Figure S7: The payoffs obtained by an honest (O_1: white) and corrupt (O_2: black circle) officer when she interacts with an apathetic (C_1: green) or conscientious (C_2: blue) or honest (C_3: red) citizens are shown above the officers in green, blue and red letters respectively. The corresponding citizen's payoff as a result of the interaction is shown below the citizens using the same colour coding.

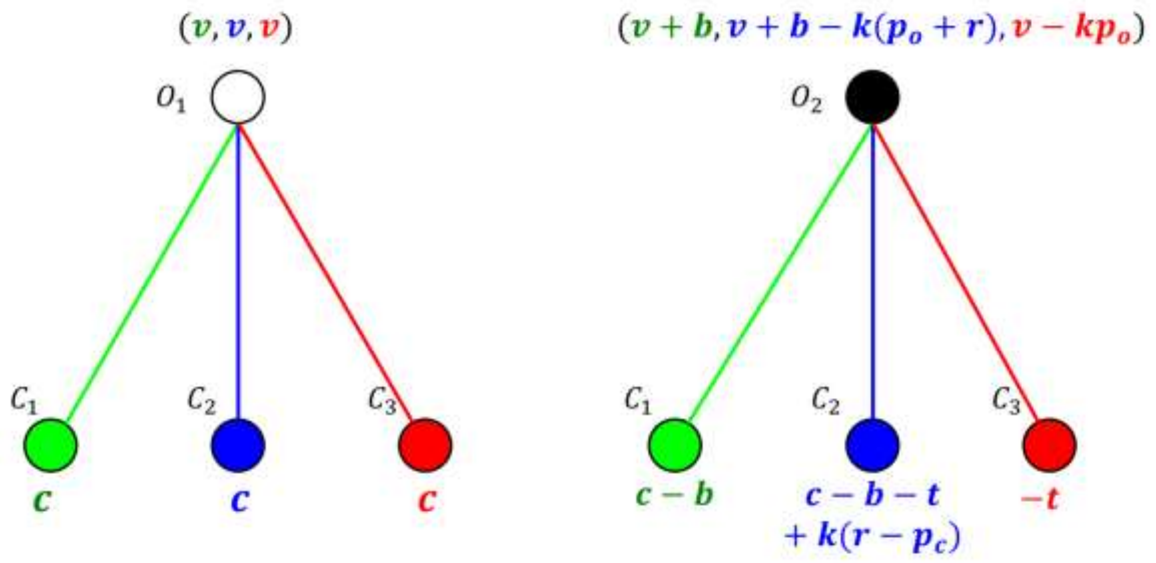

S8: Derivation of the replicator equations on an inter-dependent regular graph for the asymmetric bribery game

Consider an asymmetric 2×2 game between two categories of players. First category may choose from strategies A and B. Second category of player can choose from strategies X and Y. The payoff matrix of the game is given by:

$$M_1 = \begin{matrix} & X & Y \\ A & a' & b' \\ B & c' & d' \end{matrix} \quad (1)$$

$$M_2 = \begin{matrix} & A & B \\ X & p' & q' \\ Y & r' & s' \end{matrix} \quad (2)$$

Let p_A, p_B, p_X and p_Y be the frequencies of players with strategies A, B, X and Y respectively.

Let N_1 and N_2 be the number of each category of players in the population. Let us also assume that the population of players for each category is fixed. This implies

$$p_A + p_B = 1 \quad (3)$$

$$p_X + p_Y = 1 \quad (4)$$

Population of each category of players is connected through a network of degree k_1 and k_2. They are called intra-networks. Intra networks are also connected across each other through inter-network. The number of X and Y connected with A or B is λ_1. Similarly the number of A and B connected with X or Y is λ_2. The total number of inter-network edges is fixed:

$$\lambda_1 N_1 = \lambda_2 N_2 \quad (5)$$

Let us now assume that $q_{m|n}$ is the conditional probability that a player of strategy m has n as its neighbours. It is defined as

$$q_{m|n} = \frac{p_{nm}}{p_n} \quad (6)$$

Here p_{nm} is the number of $m-n$ edges of inter-network. From our previous assumption

$$q_{A|B} + q_{A|A} = 1 \quad (7)$$

$$q_{B|B} + q_{A|B} = 1 \tag{8}$$

$$q_{X|Y} + q_{Y|Y} = 1 \tag{9}$$

$$q_{Y|X} + q_{X|X} = 1 \tag{10}$$

$$q_{X|A} + q_{Y|A} = 1 \tag{11}$$

$$q_{X|B} + q_{Y|B} = 1 \tag{12}$$

$$q_{A|X} + q_{B|X} = 1 \tag{13}$$

$$q_{A|Y} + q_{B|Y} = 1 \tag{14}$$

Death Birth Process

According to this update rule, one random individual dies and neighbours compete for the empty location with probability proportional to their respective payoff. This happens for both categories of player (*A* & *B* and *X* & *Y*) for every update. We assume weak selection limit $w << 1$.

Suppose a player *B* dies. The fitness of player *A* & *B* connected with dead player *B* is given by:

$$f_A = 1 - w + w\lambda_1(a' q_{X|A} + b' q_{Y|A}) \tag{15}$$

$$f_B = 1 - w + w\lambda_1(a' q_{X|B} + b' q_{Y|B}) \tag{16}$$

Probability that *A* reproduces at site where *B* had died $= \dfrac{k_A f_A}{k_A f_A + k_B f_B}$. In this case population of *A* will increase by $1/N_1$.

Similarly, if player *A* dies, the fitness of *A* and *B* player connected with the dead player *A* is

$$f_A = 1 - w + w\lambda_1(a' q_{X|A} + b' q_{Y|A}) \tag{17}$$

$$f_B = 1 - w + w\lambda_1(a' q_{X|B} + b' q_{Y|B}) \tag{18}$$

Probability that *B* reproduces at site where *A* had died $= \dfrac{k_B f_B}{k_A f_A + k_B f_B}$. In this case population of *B* will increase by $1/N_2$.

$$\Pr\left(\Delta p_A = \frac{1}{N_1}\right) = p_B \sum_{\substack{k_A+k_B=k_1 \\ \lambda_X+\lambda_Y=\lambda_1}} \frac{k_1!}{k_A!\,k_B!} \frac{\lambda_1!}{\lambda_X!\,\lambda_Y!} q_{X|B}^{\lambda_X} q_{Y|B}^{\lambda_Y} q_{A|B}^{k_A} q_{B|B}^{k_B} \frac{k_A f_A}{k_A f_A + k_B f_B} \tag{19}$$

Number of *A*-*A* pairs increases by k_A and therefore p_{AA} increases by $\frac{k_A}{k_1 N_1 / 2}$. $k_1 N_1 / 2$ is the total number of edges in the intra network 1.

$$\Pr\left(\Delta p_{AA} = \frac{2k_A}{k_1 N_1}\right) = p_B \frac{k_1!}{k_A!\,k_B!} \frac{\lambda_1!}{\lambda_X!\,\lambda_Y!} q_{X|B}^{\lambda_X} q_{Y|B}^{\lambda_Y} q_{A|B}^{k_A} q_{B|B}^{k_B} \frac{k_A f_A}{k_A f_A + k_B f_B} \tag{20}$$

Number of *A*-*X* pairs increases by λ_X and therefore p_{AX} increases by $\frac{\lambda_X}{\lambda_1 N_1}$. $\lambda_1 N_1$ is the total number of edges in the inter network.

$$\Pr\left(\Delta p_{AX} = \frac{\lambda_X}{\lambda_1 N_1}\right) = p_B \frac{k_1!}{k_A!\,k_B!} \frac{\lambda_1!}{\lambda_X!\,\lambda_Y!} q_{X|B}^{\lambda_X} q_{Y|B}^{\lambda_Y} q_{A|B}^{k_A} q_{B|B}^{k_B} \frac{k_A f_A}{k_A f_A + k_B f_B} \tag{21}$$

When *A* is randomly selected for death and *B* replaces *A*, population of *A* decreases by $1/N_1$.

$$\Pr\left(\Delta p_A = -\frac{1}{N_1}\right) = p_A \sum_{\substack{k_A+k_B=k_1 \\ \lambda_X+\lambda_Y=\lambda_1}} \frac{k_1!}{k_A!\,k_B!} \frac{\lambda_1!}{\lambda_X!\,\lambda_Y!} q_{X|A}^{\lambda_X} q_{Y|A}^{\lambda_Y} q_{A|A}^{k_A} q_{B|A}^{k_B} \frac{k_B f_B}{k_A f_A + k_B f_B} \tag{22}$$

Number of *A*-*A* pairs decreases by k_A and therefore p_{AA} decreases by $\frac{k_A}{k_1 N_1 / 2}$.

$$\Pr\left(\Delta p_{AA} = -\frac{2k_A}{k_1 N_1}\right) = p_A \frac{k_1!}{k_A!\,k_B!} \frac{\lambda_1!}{\lambda_X!\,\lambda_Y!} q_{X|A}^{\lambda_X} q_{Y|A}^{\lambda_Y} q_{A|A}^{k_A} q_{B|A}^{k_B} \frac{k_B f_B}{k_A f_A + k_B f_B} \tag{23}$$

Number of *A*-*X* pairs decreases by λ_X and therefore p_{AX} decreases by $\frac{\lambda_X}{\lambda_1 N_1}$.

$$\Pr\left(\Delta p_{AX} = -\frac{\lambda_X}{\lambda_1 N_1}\right) = p_A \frac{k_1!}{k_A!\,k_B!} \frac{\lambda_1!}{\lambda_X!\,\lambda_Y!} q_{X|A}^{\lambda_X} q_{Y|A}^{\lambda_Y} q_{A|A}^{k_A} q_{B|A}^{k_B} \frac{k_B f_B}{k_A f_A + k_B f_B} \tag{24}$$

The time derivative of p_{AA} is given by:

$$\dot{p}_{AA} = \sum_{\substack{k_A + k_B = k_1 \\ \lambda_X + \lambda_Y = \lambda_1}} \frac{2k_A}{k_1 N_1} \left[\Pr\left(\Delta p_{AA} = \frac{2k_A}{k_1 N_1} \right) - \Pr\left(\Delta p_{AA} = -\frac{2k_A}{k_1 N_1} \right) \right] \tag{25}$$

On simplification gives

$$\dot{p}_{AA} = \frac{2 p_{AB}}{k_1 N_1} \left[1 + (k_1 - 1)(q_{A|B} - q_{A|A}) \right] + O(w) \tag{26}$$

The time derivative of p_A is given by:

$$\dot{p}_A = \frac{1}{N_1} \left[\Pr\left(\Delta p_A = \frac{1}{N_1} \right) - \Pr\left(\Delta p_A = -\frac{1}{N_1} \right) \right] \tag{27}$$

On simplification

$$\dot{p}_A = p_{AB} \frac{w(k_1 - 1)\lambda_1}{N_1 k_1} \left(q_{A|A} + q_{B|B} \right) \left(a' q_{X|A} + b' q_{Y|A} - c' q_{X|B} - d' q_{Y|B} \right) + O(w^2) \tag{28}$$

Similarly,

$$\dot{p}_X = p_{XY} \frac{w(k_2 - 1)\lambda_2}{N_2 k_2} \left(q_{X|X} + q_{Y|Y} \right) \left(p' q_{A|X} + q' q_{B|X} - r' q_{A|Y} - s' q_{B|Y} \right) + O(w^2) \tag{29}$$

The time derivative of p_{AX} is given by:

$$\dot{p}_{AX} = \sum_{\substack{k_A + k_B = k_1 \\ \lambda_X + \lambda_Y = \lambda_1}} \frac{\lambda_X}{\lambda_1 N_1} \left[\Pr\left(\Delta p_{AX} = \frac{\lambda_X}{\lambda_1 N_1} \right) - \Pr\left(\Delta p_{AX} = -\frac{\lambda_X}{\lambda_1 N_1} \right) \right] \tag{30}$$

On simplification

$$\dot{p}_{AX} = p_{AB} \frac{1}{N_1} \left(q_{X|B} - q_{X|A} \right) + O(w) \tag{31}$$

From the equations derived above we found that the rate of change of p_{AA} & p_{XA} is high compared to p_A & p_X . Therefore equilibrium condition $\dot{p}_{AA}=0$ & $\dot{p}_{XA}=0$

$$q_{A|A}=p_A+\frac{p_B}{(k-1)} \tag{32}$$

$$q_{A|X}=p_A \tag{33}$$

Using equations (7) – (14), (28), (32) and (33) we have

$$\dot{p}_A=\frac{w(k_1-2)}{N_1(k_1-1)}p_A p_B\left[\left(a'-b'-c'+d'\right)p_X+(b'-d')\right] \tag{34}$$

Imitation Update

In imitation update rule, a random individual (focal individual) is chosen to update its strategy. The individual retains its strategy or imitate the strategy of one of its neighbor (role model) with probability proportional to the fitness of role model.

Let the fitness of a *B* player with $\lambda_X, \lambda_Y, k_A$ and k_B number *X, Y, A* and *B* neighbours respectively be

$$f_o=1-w+w(\lambda_X c'+\lambda_Y d') \tag{35}$$

Probability that a *B* player imitates a neighbor with *A* strategy is given by

$$\frac{k_A f_A}{k_A f_A+k_B f_B+f_o} \quad \dot{p}_{AX}=p_{AB}\frac{1}{N_1}\left(q_{X|B}-q_{X|A}\right)+O(w) \tag{36}$$

Similarly, let the fitness of a *A* player with $\lambda_X, \lambda_Y, k_A$ and k_B number of *X, Y, A* and *B* neighbours respectively is given by

$$g_o=1-w+w(\lambda_X a'+\lambda_Y b') \tag{37}$$

Probability that *A* imitate *B*'s strategy is

$$\frac{k_B f_B}{k_A f_A + k_B f_B + g_o} \tag{38}$$

Similar to the case as described in 'death birth process' section the time derivative of p_{AA} is given by:

$$\begin{aligned}\dot{p}_{AA} &= \sum_{\substack{k_A+k_B=k_1\\ \lambda_X+\lambda_Y=\lambda_1}} \frac{2k_A}{k_1 N_1}\left[\Pr\left(\Delta p_{AA} = \frac{2k_A}{k_1 N_1}\right) - \Pr\left(\Delta p_{AA} = -\frac{2k_A}{k_1 N_1}\right)\right] \\ &= \sum_{\substack{k_A+k_B=k_1\\ \lambda_X+\lambda_Y=\lambda_1}} \frac{2k_A}{k_1 N_1}\left[p_B \frac{k_1!}{k_A!\,k_B!}\frac{\lambda_1!}{\lambda_X!\,\lambda_Y!} q_{X|B}^{\lambda_X} q_{Y|B}^{\lambda_Y} q_{A|B}^{k_A} q_{B|B}^{k_B} \frac{k_A f_A}{k_A f_A + k_B f_B + f_o} \right. \\ &\qquad \left. - p_A \frac{k_1!}{k_A!\,k_B!}\frac{\lambda_1!}{\lambda_X!\,\lambda_Y!} q_{X|A}^{\lambda_X} q_{Y|A}^{\lambda_Y} q_{A|A}^{k_A} q_{B|A}^{k_B} \frac{k_B f_B}{k_A f_A + k_B f_B + g_o}\right]\end{aligned} \tag{39}$$

Under weak selection limit ($w << 1$) the above expression simplifies to

$$\dot{p}_{AA} = \frac{2 p_{AB}}{(k_1+1)N_1}\left[1 + (k_1 - 1)(q_{A|B} - q_{A|A})\right] + O(w) \tag{40}$$

The time derivation of p_{AX} is same as given in equation (30). This equation for imitation update simplifies to

$$\dot{p}_{AX} = p_{AB}\frac{1}{N_1}\frac{k_1}{k_1+1}\left(q_{X|B} - q_{X|A}\right) + O(w) \tag{41}$$

Time derivative of p_A for imitation update is given by

$$\begin{aligned}\dot{p}_A &= \frac{1}{N_1}\left[\Pr\left(\Delta p_A = \frac{1}{N_1}\right) - \Pr\left(\Delta p_A = -\frac{1}{N_1}\right)\right] \\ &= \sum_{\substack{k_A+k_B=k_1\\ \lambda_X+\lambda_Y=\lambda_1}} \frac{1}{N_1} p_B \frac{k_1!}{k_A!\,k_B!}\frac{\lambda_1!}{\lambda_X!\,\lambda_Y!} q_{X|B}^{\lambda_X} q_{Y|B}^{\lambda_Y} q_{A|B}^{k_A} q_{B|B}^{k_B} \frac{k_A f_A}{k_A f_A + k_B f_B + f_o} \\ &\qquad - \frac{1}{N_1} p_A \frac{k_1!}{k_A!\,k_B!}\frac{\lambda_1!}{\lambda_X!\,\lambda_Y!} q_{X|A}^{\lambda_X} q_{Y|A}^{\lambda_Y} q_{A|A}^{k_A} q_{B|A}^{k_B} \frac{k_B f_B}{k_A f_A + k_B f_B + g_o}\end{aligned} \tag{42}$$

On simplifying the above equation under weak selection limit ($w << 1$) gives

$$\dot{p}_A = p_{AB} \frac{wk_1}{N_1(k_1+1)^2}\left[2+(k_1-1)\left(q_{A|A}+q_{B|B}\right)\right]\left(a'q_{X|A}+b'q_{Y|A}-c'q_{X|B}-d'q_{Y|B}\right)+O(w^2) \quad (43)$$

Using the fact that the rate of change of p_{AA} & p_{XA} is high compared to p_A

$$\dot{p}_A = \frac{wk_1(k_1-2)(k_1+2)}{N_1(k_1-1)(k_1+1)^2} p_A p_B \left[\left(a'-b'-c'+d'\right)p_X+(b'-d')\right] \quad (44)$$

Birth Death Process

In birth death process, an individual is first chosen for reproduction with probability proportional to its fitness. This individual replaces a randomly chosen neighbour which is chosen for death.

The probability that an *A* player who has λ_X, λ_Y, k_A and k_B number *X, Y, A* and *B* neighbours is selected for reproduction is proportional to

$$p_A \frac{k_1!}{k_A!\,k_B!}\frac{\lambda_1!}{\lambda_X!\,\lambda_Y!} q_{X|A}^{\lambda_X}\, q_{Y|A}^{\lambda_Y}\, q_{A|A}^{k_A}\, q_{B|A}^{k_B}\left[1-w+w(\lambda_X a'+\lambda_Y b')\right] \quad (45)$$

Similarly if *B* is selected for reproduction then the probability is proportional to

$$p_B \frac{k_1!}{k_A!\,k_B!}\frac{\lambda_1!}{\lambda_X!\,\lambda_Y!} q_{X|B}^{\lambda_X}\, q_{Y|B}^{\lambda_Y}\, q_{A|B}^{k_A}\, q_{B|B}^{k_B}\left[1-w+w(\lambda_X c'+\lambda_Y d')\right] \quad (46)$$

When is selected for reproduction and *B* is selected for death the number of *AA* pairs increases by $\left[1+(k_1-1)q_{A|B}\right]$

$$\begin{aligned}\dot{p}_{AA} = \sum_{\substack{k_A+k_B=k_1\\ \lambda_X+\lambda_Y=\lambda_1}} \Bigg[& \frac{k_B}{k_1N_1} p_A \frac{k_1!}{k_A!\,k_B!}\frac{\lambda_1!}{\lambda_X!\,\lambda_Y!} q_{X|A}^{\lambda_X}\, q_{Y|A}^{\lambda_Y}\, q_{A|A}^{k_A}\, q_{B|A}^{k_B}\left[1-w+w(\lambda_X a'+\lambda_Y b')\right]\left[1+(k_1-1)q_{A|B}\right] \\ & -\frac{k_A}{k_1N_1} p_B \frac{k_1!}{k_A!\,k_B!}\frac{\lambda_1!}{\lambda_X!\,\lambda_Y!} q_{X|B}^{\lambda_X}\, q_{Y|B}^{\lambda_Y}\, q_{A|B}^{k_A}\, q_{B|B}^{k_B}\left[1-w+w(\lambda_X c'+\lambda_Y d')\right]\left[(k_1-1)q_{A|A}\right]\Bigg]\end{aligned} \quad (47)$$

On simplifying the above equation under weak selection limit ($w << 1$) gives

$$\dot{p}_{AA} = \frac{p_{AB}}{N_1}\left[1+(k_1-1)(q_{A|B}-q_{A|A})\right]+O(w) \quad (48)$$

For the rate of change of AX we can similarly write

$$\begin{aligned}\dot{p}_{AX} = \sum_{\substack{k_A+k_B=k_1 \\ \lambda_X+\lambda_Y=\lambda_1}} \Bigg[& \frac{k_B}{k_1 N_1} p_A \frac{k_1!}{k_A!\,k_B!} \frac{\lambda_1!}{\lambda_X!\,\lambda_Y!} q_{X|A}^{\lambda_X} q_{Y|A}^{\lambda_Y} q_{A|A}^{k_A} q_{B|A}^{k_B} \left[1-w+w(\lambda_X a'+\lambda_Y b')\right]\lambda_1 q_{X|B} \\ & -\frac{k_A}{k_1 N_1} p_B \frac{k_1!}{k_A!\,k_B!} \frac{\lambda_1!}{\lambda_X!\,\lambda_Y!} q_{X|B}^{\lambda_X} q_{Y|B}^{\lambda_Y} q_{A|B}^{k_A} q_{B|B}^{k_B} \left[1-w+w(\lambda_X c'+\lambda_Y d')\right]\lambda_1 q_{X|A} \Bigg]\end{aligned} \quad (49)$$

On simplifying under weak selection limit ($w<<1$) gives

$$\dot{p}_{AX} = p_{AB}\frac{k_1\lambda_1}{N_1}\left(q_{X|B}-q_{X|A}\right)+O(w) \quad (50)$$

Finally, the time derivative of p_A is given by:

$$\dot{p}_A = \frac{w(k_1-2)}{N_1(k_1-1)} p_A p_B \left[\left(a'-b'-c'+d'\right)p_X + (b'-d')\right] \quad (51)$$
